# Evaluation of an intervention to improve the safety of medication therapy via HIT-supported interprofessional cooperation in long-term care – a mixed method study

**DOI:** 10.1186/s12913-022-08562-6

**Published:** 2022-10-03

**Authors:** Maria Magdalena Schreier, Stefan Pitzer, Johanna Katharina Dellinger, Dagmar Schaffler-Schaden, Jürgen Osterbrink, Maria Flamm

**Affiliations:** 1 Institute of Nursing Science and Practice, Paracelsus Medical University, Salzburg, Austria; 2 Institute of General Practice, Family Medicine and Preventive Medicine, Paracelsus Medical University, Salzburg, Austria

**Keywords:** Long-term care, Interprofessional cooperation, Drug therapy, Health information technology (HIT), Evaluation research

## Abstract

**Background:**

In order to ensure the provision of appropriate and safe medication therapy in long-term care, close interprofessional cooperation and high levels of expertise are required. Online digital documentation and communication technology facilitate this process. The aim of the present study (sub-study 2 of the SiMbA-Study) was to evaluate a three-part health information technology (HIT) driven intervention on medication therapy safety in Austrian nursing homes (NHs) regarding its usefulness, practicability and implementation in routine care.

**Methods:**

A concurrent embedded mixed-methods design was conducted to evaluate the intervention. Data was collected via expert interviews, focus group discussions and quantitative survey of general practitioners, nurses, and pharmacists in 3 NHs. Usefulness and effectiveness of the intervention were investigated through summative evaluation. Formative evaluation was utilized to gain insights regarding features and factors of the implementation process necessary to a successful integration in routine care.

**Results:**

The sample comprised general practitioners, pharmacists, and nurses. 23 participants were interviewed, of which 17 participated in the focus group discussions and completed the quantitative Survey. All components of the intervention were deemed to be useful and effective. Effort and benefit of using health information technology were well balanced. Implementation success was mainly attributed to socio-normative factors.

**Conclusions:**

The implementation of HIT-based measures can be effective but is prone to various pitfalls that are highlighted in the study. A critical challenge for successful implementation is the combination of both, ensuring its prerequisites, while anticipating new problems that arise from HIT-integration on the one hand and changes in interprofessional cooperation on the other.

**Trial registration:**

DRKS Data Management, ID: DRKS00012246. Registered 16.05.2017 – Retrospectively registered.

**Supplementary Information:**

The online version contains supplementary material available at 10.1186/s12913-022-08562-6.

## Background

Medication safety among older adults who require long-term care is a very important goal because medication errors can result in polypharmacy [1, 2], adverse drug events, and drug-drug interactions [3–6]. General practitioners (GPs), pharmacists, and nurses should cooperate to meet the high standard required for safe provision of drug therapy to older adults [4, 7–9]. According to Tariq et al. (2013) and Tariq et al. (2016) [10, 11], three major factors pose threats to medication safety. The first factor is complicated prescriptions and documentation procedures. The second factor pertains to the manner in which information about changes in drug therapy is exchanged between the professional groups who are involved in the management of drug therapy: In nursing home practice, such information is primarily exchanged through telephone and facsimile communication, which can adversely impact the reliable exchange of information. Insufficient cooperation between GPs, pharmacists, and nurses represents a third significant threat to medication safety [10, 11].

The drug therapy provided in nursing homes (NHs) can be improved by implementing a variety of measures: relying on the specialist expertise of the actors involved, careful prescribing by GPs, medication checks by pharmacists, and better information transfer and case conferences between GPs, pharmacists, and nurses [12]. Health information technology (HIT) can be used to meet the high demands of medication safety because it can improve supply security, teamwork, and cooperation between professional groups [13, 14]. However, the following are essential prerequisites for its appropriate application: (a) the tools should be user friendly, (b) the interactions between individuals, technology, organizations, tasks, and the environment should be taken into account within the respective care contexts, and (c) the users of the technical tools should be well trained [14].

Against this background, the SiMbA-study (“Sicherheit der Medikamententherapie bei AltenheimbewohnerInnen”, Safety of medication therapy in nursing home residents) implemented an interprofessional intervention among GPs, pharmacists, and nurses, designed to improve medication safety in NHs whilst taking into account the aforementioned obstacles and facilitators of utilizing HIT mentioned above. It consisted of the following three components, which were adapted to the reality of care of the participating NHs [15, 16]:

1. Drug therapy safety training: All healthcare professionals completed a three-part-training about medication safety in older adults. The process started with an interprofessional workshop (3 h) of the participating GPs, pharmacists, and nurses led by medication safety experts from each of the three professions. It addressed the basics of medication safety, legal issues regarding adverse drug events, the duties of each profession and the many opportunities of interprofessional risk management in residential care. The workshop was followed by three profession-specific online learning modules (20–45 min per module) which revisited and deepened the foundations laid in the workshop. Finally, all professionals had to review case examples of complex drug therapy and pass a multiple-choice test. All participants had the opportunity to interact with the experts per mail until the final test. The first component of the intervention concluded with a further interprofessional meeting to reflect the preceding process and prepare the utilization of the training in the second and third component.
2. Implementation of the interactive communication and documentation tool SiM-Pl (SiMbA-Platform): The HIT-tool SiM-Pl was designed as an expansion of the nursing home’s electronic health record (EHR) for mobile as well as stationary use. It complemented the EHR with a new messaging system for interprofessional communication, a nursing assessment to oversee symptoms related to current and changed medication [17] as well as a medication review form [18]. Professionals outside the NHs (GPs and pharmacists) were able to access the EHR and the messaging system via provided tablets and a security-token. In this way, GPs gained access from their office and pharmacists had access to the EHR for the first time.
3. Drug therapy check process: Healthcare professionals had to participate in standardized medication review and monitoring (for full details, see the study protocol [15]). First, GPs verified the entries of drugs and diagnoses in the EHR of their patients. Second, pharmacists reviewed the medication of each participant via the medication review form. They repeated their review at least once before post-test. Nurses had the task to monitor and record adverse changes in health status of each NHR at least every week and after each medication change. GPs were provided with the current results of the medication review and monitoring processes when logging into the SiM-Pl. The messaging system allowed interprofessional communication (e.g. requests regarding individual medication checks) at any time of the drug therapy check process.

The SiMbA-study comprised two sub-studies, both examining this three-part intervention:

Sub-study 1 tested the effectivity of the intervention in improving a patient-related outcome, the appropriateness of the drug therapy as measured by the medication appropriateness index (MAI). The results of this sub-study are already published and confirmed the hypothesized relevant improvement (MAI-reduction ≥ 3) in drug therapy in patients with comparatively poor medication appropriateness (i.e., MAI ≥ 23; mean difference of MAI-change compared to the control group was 6.8) [16].

**Fig. 1:**
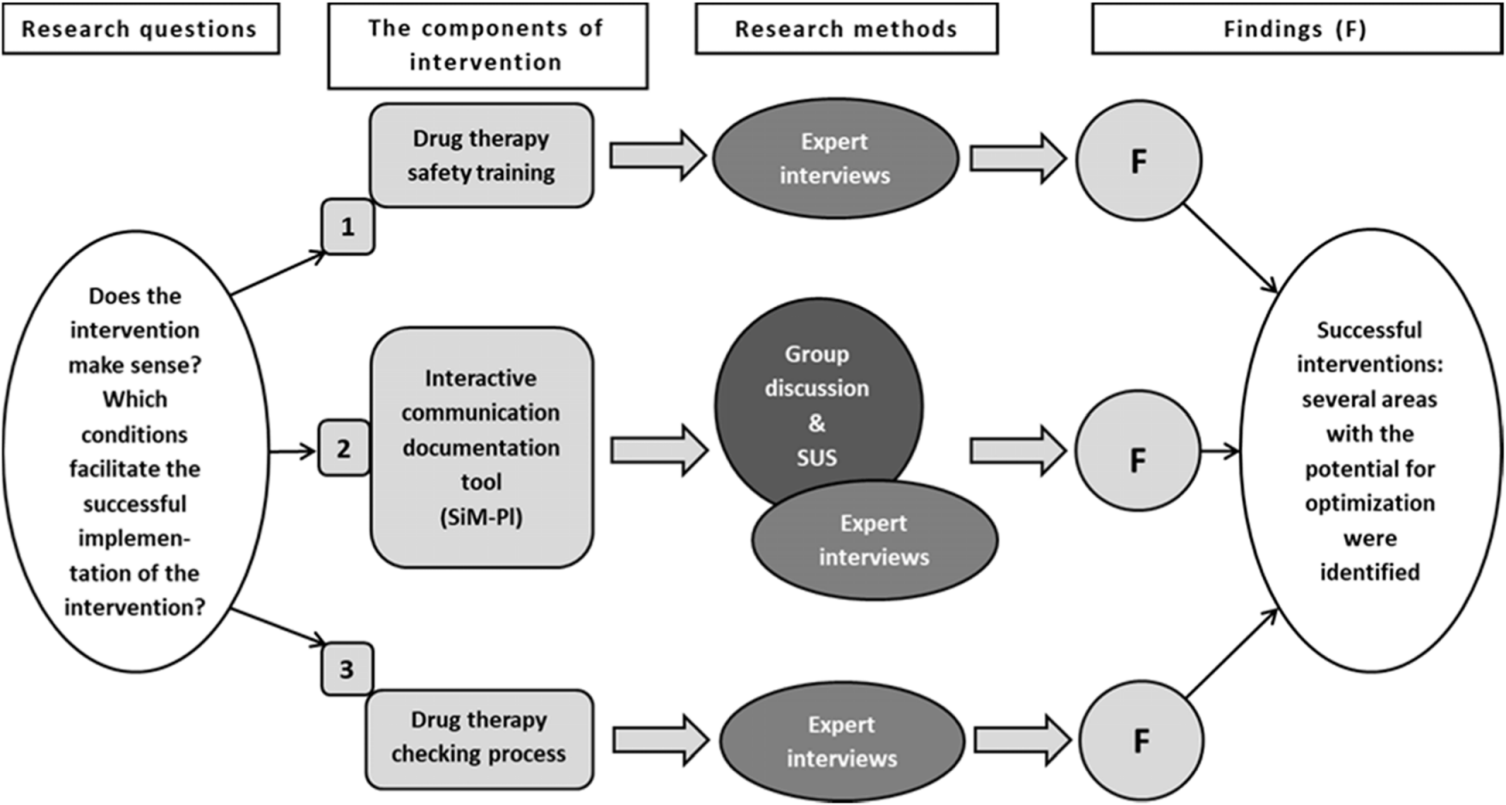
An overview of the study, including the evaluation of the three components of the SiMbA intervention using different research methods

Sub-study 2 is the subject of the present article. It was conducted to generate insights regarding features and factors important to integrating the intervention into routine care. It was therefore the aim of the study to examine GPs’, pharmacists’, and nurses’ perspectives (1) on the interventions’ usefulness, practicability, and impact on work processes, and (2) on how this intervention can be successfully implemented in daily practice. Accordingly, the following research questions were pursued. First, how do the participating GPs, pharmacists, and nurses evaluate the appropriateness of the three components of the intervention (i.e., does the intervention make sense, see Fig. 1)? Second, which conditions facilitate the successful implementation of the intervention?

## Methods

### Study design, setting and selection of participants

A concurrent embedded mixed-methods design was conducted to evaluate the intervention. ‘Embedded’ refers to the integration of quantitative methods into a qualitative methodology framework, or vice versa, to provide enriched insights or understanding into the phenomena of interest [19, 20]. The embedded approach was confined to the evaluation of the interactive communication and documentation tool whilst other elements of the intervention were evaluated through a qualitative approach (see Fig. 1). The intervention was implemented as part of sub-study 1, which took place between 2016–2018 in 6 Austrian NHs from which 3 were assigned to the intervention group (see [16] for further details on the selection of NHs). Sub-study 2, the study presented here, took place in 2018 after the follow-up phase of the intervention.

The sampling frame was predefined by the intervention: Only professionals directly engaged in the intervention were eligible to participate, i.e. GPs primarily responsible for the prescription and monitoring of their patients’ medication, nurses who distribute medication and monitor intake and functioned as coordinating nurse, and community pharmacists providing the prescribed medication in the 3 NHs of the intervention group. We aimed to include all 11 GPs, 4 pharmacists and 12 coordinating nurses that took part in the intervention.

Both summative and formative evaluation were conducted. Summative evaluation was applied to investigate the usefulness and effectiveness of the SiMbA-intervention. Formative evaluation was utilized to explore required modifications to the intervention and its implementation process necessary to a successful integration in routine care [21–24]. Semi-structured expert interviews and focus group discussions among GPs, pharmacists, and nurses as well as a standardized quantitative survey on the usability of the interactive communication and documentation tool (i.e., SiM-Pl) were conducted. Expert interviews were chosen to record as many different and individual experiences with the three components of the intervention as possible. Particular attention was paid to SiM-Pl because of its central importance to the exchange of information between professional groups who are involved in the provision of drug therapy. Therefore, it was more comprehensively evaluated through focus group discussions and using a quantitative usability measure (i.e., the System Usability Scale; SUS) [25].

Considering the small sampling frame (especially regarding the number of pharmacists) and assurance of anonymity to all participants, a juxtaposition of profession-specific perspectives of GPs, pharmacists and nurses as well as a comparison of nursing homes was not intended in data analysis.

Figure 1 presents an overview of the study, including the evaluation of the three components of the SiMbA-intervention using different research methods.

### Research methods used: a detailed description

#### Semi-structured expert interviews

Semi-structured expert interviews are conducted with experts who possess in-depth domain-specific knowledge [26]. Pretested interview schedules, which include targeted questions about specific topics, were used to facilitate delimitation and in-depth exploration [27, 28] (see Additional file 1 for interview schedule).

To overcome geographical barriers, enhance time efficiency, and offer greater flexibility in the appointments that were scheduled with the professional groups, interviews were carried out via telephone. Telephone interviews are suitable for use when all the participants are familiar with the topic of the survey [29], and this was the case with the participants of the present evaluation study. The possible disadvantages of telephone interviews (e.g., poorer control over the interview environment, when compared to face-to-face interviews) were addressed conceptually (see Additional file 2, Tab. 1).

Interviews were thematically structured along the three components of the intervention. The participants were questioned about their impressions of and experiences with the intervention as well as the cooperation and communication between the different professional groups. Probes were used to directly clarify unclear and ambiguous statements. Specifically, the interviewers provided clarifying details, examples, or confirmations whenever they were deemed helpful or necessary [29, 30].

Trained interviewers (1 male, 1 female) with master’s degree and experience in nursing science, who were informed about but not involved in the project and were unknown to the participants carried out the interviews 6 weeks before the planned focus group discussions. The tape recordings of the interviews, each of which lasted for approximately 30 min, were transcribed, semantically smoothed, and subjected to content analysis using the software MAXQDA [31, 32]. Content analysis was conducted by the first author. Initially, deductive analysis was undertaken in accordance with the topics of the interview schedule to generate the main codes. Subsequently, inductive content analysis was conducted and the phenomena that were identified from the interview data were grouped into subcategories. This process resulted in a higher differentiation of the code system with each passage; the research question was constantly borne in mind, and this informed the interpretation of the transcribed interview recordings [31, 33]. After the completion of content analysis, the final codes were compared against the original material and the members of the research team resolved any disagreements through discussions. The expert interviews were subjected to an additional deductive analysis of the balance between efforts and benefits associated with the intervention.

#### Focus group discussions

To evaluate the hardware (iPad) and software (SiM-Pl) of the interactive documentation and communication tool that had been used in the SiMbA-study, informal interactive exchanges between the experts of the intervention group were facilitated by conducting a focus group discussion. The mutual influence of the participants of focus group discussions can adversely affect (e.g., social desirability, overemphasis of the opinions of the dominant participants) the disclosed content. However, focus group discussions can also result in changes in arguments and statements [30, 34], which tend to be reflective of a more precise and concrete definition. Thus, this research method was expected to yield valuable information about the suitability of the interactive communication and documentation tool.

The successful use of interactive systems depends on their usability [35]. Therefore, affording the users the opportunity to comment on and evaluate the usability of an interactive product such as the SiM-Pl increases the chances of successfully implementing it [35]. The pragmatic, experience-oriented, and hedonic (emotionally pleasing) aspects of interactive products influence their acceptance and use among individuals. Accordingly, a discussion schedule was developed based on the hedonic-pragmatic model that has been proposed by [35] (for details, see Additional file 2, Tab. 2).

Since moderating a group discussion is a very demanding task [34], two members of the research team served as moderators. The discussion schedule was used to refocus the discussion whenever necessary [27, 34]. In order to avoid imposing a rigid structure on the discussions, only a brief description of each topic was presented to the participants; probes were used to provide additional clarifications only when necessary (see Additional file 1 for focus group schedule).

The group discussion took place in a meeting room at the location of the participating NHs. Over the course of the discussion, the topics being discussed and the core statements that were generated by the participants were visualized using a flip chart and validated [34].

A third member of the research team transcribed the proceedings of the interview using a laptop. Subsequently, the resulting data were subjected to deductive and inductive content analysis led by the first author [31, 33, 36, 37]. The analysis of the group discussion, which was oriented towards Mayring [36], was aligned with the deductive-inductive analysis procedure according to Kuckartz [32, 37] in the course of the processing. First, deductive analysis was used to structure the content in accordance with the topics that were covered by the discussion schedule; next, summarizing inductive content analysis was undertaken. The codes were compared (i.e., based on their content) and combined into thematic categories. The members of the research team continuously discussed and reviewed these decisions throughout the analytic process.

#### System usability scale

The mixed-methods part of this study is the two-part investigation of the usability of the SiM-Pl. It was done both in the focus group discussions via qualitative questioning (for details, see Additional file 1: evaluation of the program SiM-Pl) and via using the system usability scale (SUS). The SUS is a standardized, easy-to-use, and well-established scale to assess the usability of technical devices, computer systems (hardware and software), or interactive communication products [24, 38–41]. It has been shown to be a valid measure not only of subjective usability, but also of actual usability [42], adding to the perspective gained from the focus group interviews.

Immediately prior to the focus group discussions, participants responded to a pen-and-paper version of the SUS after they were provided with brief instructions. A German translation of the SUS was used (cf. Rügenhagen & Rummel, 2015 [43]). The term “system,” which is used in the original scale, was replaced with terms that were familiar to the interviewees. Descriptive statistics were computed using Excel, in accordance with the instructions that have been provided by Brook (1996), Brook (2013) and Bangor et al. (2009) [25, 38, 40]. The result of the SUS was merged with the usability evaluation from the focus group discussions via qualitizing the SUS-Score as suggested by the adjective ratings from Bangor et al. (2009) [38].

## Results

### Sample

Twenty-three participants were interviewed, of which 17 participated in focus group discussions and completed the SUS. From each NH at least one GP, nurse and pharmacist was interviewed. In each of the three focus groups (one per NH) all three professions were involved. Focus group size was 4 to 8 and discussion duration 1 to 1.5 h. Some GPs, pharmacists, and nurses were unable to participate in the evaluation because of illnesses and time constraints. Participation rates in the interviews and focus group discussions were 85% and 63%, respectively. Table 1 shows the sample profile. For reasons of anonymity, gender is only assigned to the overall group of participants, not to the professions.

**Table 1:** Profile of the sample used to evaluate the SiMbA-intervention; Notes: *n* = sample size (out of potential participants), SUS = System usability scale, GP = General practitioner

	**Expert interviews**	**Focus group discussion and SUS**
Sample (n)	*n* = 23/27 *(excluded: n* = *1; reason: defective recording)*	*n* = 17/27 *(excluded: n* = *1; reason: missing data)*
Groups of professionals	GPs: *n* = 9/11 Pharmacists: *n* = 3/4 Nurses: *n* = 11/12	GPs: *n* = 6/11 Pharmacists: *n* = 2/4 Nurses: *n* = 9/12
Gender	Men = 4; Women = 19	Men = 3; Women = 14

### Results relative to intervention component

#### Drug therapy safety training

The medication safety training was perceived as an interesting, stimulating, and well-assisted intervention component. It helped the participating GPs, pharmacists, and nurses be more attentive and mindful when providing drug therapy.

You just think a little more... Through that, you’re just a little more conscious and think even more about it – observe, perhaps, even more closely. (ID22)

Medication interactions are already, let’s say not new to me, but, still, they’ve come to my awareness again, so that I have been paying more attention to them again. (ID20)

The use of both face-to-face interactions and online sessions in the medication safety training was perceived to be useful. It facilitated interactive exchange between the professional groups. Specifically, lectures could be provided during classroom events, and individualized, flexible learning could be promoted through online sessions.

The SiMbA training was useful to me in so far as the communication with the individual groups was possible: so, with the SiMbA team (. . .) and, then, with the nursing staff, with the pharmacy, and, of course, among the colleagues as well. Of course, this also made it possible to experience this network. (ID13)

Well, it was pleasant for me because you can do it [note: online training] when you have time and not only when you have the meeting. (ID2)

#### Interactive communication and documentation tool SiM-Pl

The participants reported positive user experiences with the SiM-Pl. All the professional groups mentioned that its use had markedly improved the ease of their work. The SiM-Pl and the drug therapy checking process significantly facilitated the processes of medical care and led to a high degree of safety in the medication instructions of GPs.

*. . . the platform – the program is great, yes! Well, it’s clear, it’s fast, you have everything at hand . . . even if there’s a problem on Friday afternoon, and I prescribe something, then, I can enter it online as well. And just for (. . .) security. Because (. . .), if you only pass something on over the phone, it can lead to (. . .) misinformation as well. You can also discuss something over the phone in advance and, then, record it in writing. (ID11)*

*As I said, for me, it’s simply a more compact way of working, because I don’t have to work through it in several phases, but I can do it with a mouse click on one thing . . . So, in terms of work and time, it is certainly optimal. It’s certainly a time saver . . . I don’t think the system is bad in itself, because, if I get there as a substitute doctor, for example, it’s just there to take and consult. (ID22)*

The following obstacles/barriers hindered the use of the tool: a personal dislike for computer work or working on a tablet, and the shortcomings pertaining to the usability of individual components of the tool. Pertinent examples include a lack of or complicated access to other relevant programs (e.g., medication lists, interaction programs) and a lack of links to GP documentation programs.

*. . . for the younger ones, the tablet [note: iPad] is a bit of an incentive. Yes, they just like to work on them; that’s kind of fun for them, yes. How to appeal to the older generation? What kind of incentive should there be? I don’t know. It’s all a time factor, of course. They say, well, I’m three times faster on the computer; we don’t have much time anyway. Some of them just don’t want to deal with it. And I think you have to accept that somehow, it’s not for everyone. (ID4)*

The message system that is integrated into the SiM-Pl was regarded as a helpful alternative to telephone and facsimile communication, but it was used only minimally because of its complex dial-in modalities. The incoming messages were visible only after the system had been accessed, and this feature was impractical. Further, the options to send and receive messages and attach files were functionally limited.

*Well, I think if the GP really answer[s] directly to these inquiries, . . . if he sees in the surgery/practice that there is a message and, then, also answers it, then, that is, of course, a big help, because, then, we get our problems solved promptly, and he immediately enters it in the nursing documentation, and I have a prescription without needing a fax or anything else. And he can write what is prescribed directly into our system. If he’s nice, he’ll enter the medication right away, and, then, I’ve actually provided legal security for all-round care! Then, of course, that is also the ideal. (ID7)*

*I say it’s useful, definitely. I’ve looked in a few times, not so often; it’s a bit complicated. You’d still have to (. . .). I’m not a computer specialist, and I don’t know how to simplify it. But it’s almost [like] a personal exchange is easier. (ID18)*

The interviewees ascribed a high potential for improvement in medication safety to the HIT tool. According to them, this not only improved the quality of life of the residents because of precise drug regulation but also reduced costs by avoiding polypharmacy.

*. . . the reduction of medication and the improved quality of life for the residents. And, at the bottom line, by way of the medicine reduction also, a cost reduction for the healthcare system . . . I think, on the one hand, the cost factor, and, on the other hand, . . . for us, the security as well. That we simply know, we produce fewer interactions, produce fewer side effects, and comes cheaper. (ID11)*

The interview data were subjected to an additional analysis of the balance between efforts and benefits in terms of the work and time that are required to conduct the SiMbA-intervention using the SiM-Pl. Four main categories with their subcategories were identified, quantified and assigned to efforts and benefits: We found that 87% (27/31) of the responses regarding the use of SiM-Pl judged it to be facilitative and timesaving. Further, 44% (8/18) of the responses about iPad usage indicated that using the SiM-Pl on their iPads simplified work and saved time because it allowed access to the documentation system anywhere and at any time. The editing option that was available to GPs ensured the immediate establishment of the legal validity of medical orders. On the other hand, 56% (10/18) of the responses regarding iPad usage concerned difficulties primarily because of an unstable wireless local area network (WLAN) connection and the resulting unreliable or impossible access to the SiM-Pl. Additionally, 53% (8/15) of the responses concerning the integrated messaging system implied that it facilitated their work and saved time, whilst 47% pointed toward additional demands like the necessity to constantly check messages to ensure timely access to important information. 78% (47/60) of the responses about the continuation or expansion of SiM-Pl usage were in support of continuation or expansion. The main reasons were the simplification of work procedures by providing flexible access to the documentation system and the enhancement of medication therapy monitoring. However, 21% (13/69) of the responses indicated that continuation or expansion is not worthwhile unless the software that GPs use in their practice is linked to the SiM-Pl; without this link, its use necessitates time-consuming double documentation by GPs.

Results derived from focus group discussion data pertained to two topics: (a) the practicability of the HIT solution and (b) its impact on work processes and daily routines in professional and personal life.

- *Practicability of SiM-Pl:* The hardware and software of the SiM-Pl were evaluated positively; they were perceived to be up to date in terms of functionality, handling, and equipment. The following aspects were negatively evaluated: the limited operating functions of the older iOS versions (i.e., an absence of the Split View/Slide Over option which permits the user to simultaneously work on two applications), hygienic concerns relating to the use of an iPad while performing patient-oriented tasks, and the unreliable functionality that results from an unstable WLAN.
- *Impact on work processes and daily routines in professional and personal life:* Since the SiM-Pl enables flexible online access to residents’ records, the participants were motivated to be more involved in medication safety. The SiM-Pl was perceived to have contributed to the optimization of various medication management processes, ranging from prescribing medications to medication analysis and administration. The SiM-Pl was perceived to have had a positive effect on work processes, the organization of daily routines within and outside the NHs, and the balance between work and leisure among the participating GPs and pharmacists. Additionally, preparations for medical visits as well as the work of substitute doctors were perceived to have been facilitated by simple access to patient records, which were provided by the SiM-Pl. The benefits of the immediate establishment of the legal validity of GPs’ medications to nurses, which were mentioned during the interviews, were also reported during the focus group discussion. In contrast, flexible access to the documentation made them feel as though they were constantly available, and this resulted in demotivation and negative feelings. The GPs agreed that the lack of a link between the SiM-Pl and the software that they use in their practice and a personal lack of affinity for computer work were core barriers that hindered their use of the tool.

**Fig. 2:**
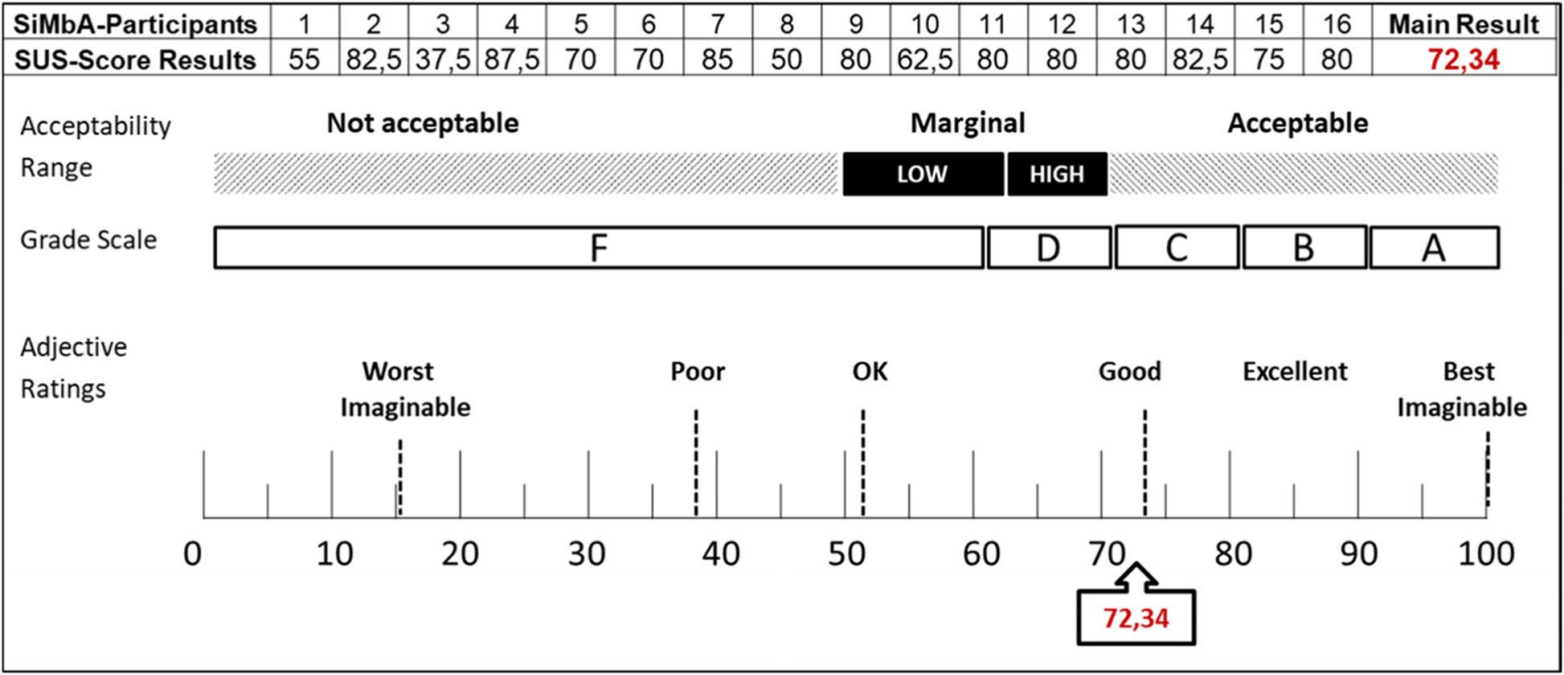
Results for the evaluation of the usability of the SiM-Pl using the SUS (Figure based on Bangor et al. 2009); Note: SUS = System usability scale

The evaluations of the usability of the SiM-Pl, which were derived using the SUS, were indicative of good or acceptable usability, and the corresponding score was 72.3% (cf. Fig. 2). This finding concurs with the conclusions drawn based on the data generated through expert interviews and focus group discussion.

#### Drug therapy check process

The participants stated that, over the course of the SiMbA-study, interprofessional cooperation (which was perceived to be satisfactory even at the beginning of the study) improved considerably. The drug therapy check process functioned well, but participants spotted an opportunity for improvement through a closer cooperation between GPs and pharmacists. For nursing staff the standardized process led to a closer involvement in drug therapy, and this facilitated the work of GPs. Close interprofessional cooperation was perceived to have enhanced medication safety, and the participants wished to strengthen their cooperation, especially with regard to the involvement of pharmacists.

A positive influence, I find, yes. I found it very positive how the feedback from the pharmacist and also the feedback from the nursing staff... made work much easier for us doctors. (ID5)

Participation in the SiMbA-study led the participants to reflect upon medication safety and contributed to the reduction of professional prejudice. Further, the various professional groups approached each other more frequently over the course of the study, gained mutual trust, perceive themselves rather as partners than opponents and developed a greater tolerance for mutual feedback.

*And trust is simply strengthened. And that you are not seen as an opponent if you say: "Take a look at the medication" or: "Is that okay?". There has been more teamwork and you no longer see them [note: other professions] as an enemy. In the beginning, when the pharmacy said something to the doctors, the doctors said, "We already know!" and that's actually gone now. Well, those who are on board [note: take part in SiMbA], they work well together (…) You no longer see them [note: other professions] as an opponent, but as a partner. (ID17)*

*That you promote awareness, in general, that everyone is aware of it (. . .), all professional groups - that you work together. . . The project, yes, was very positive for me. And it simply increases awareness, I think . . . all this monitoring, that you check it more often. (ID10)*

The interview data revealed that the exchanges between the different professional groups still primarily relied on communication via telephone. According to the interviewees, this entails a high risk of miscommunication, especially with regard to the prescription of medications by GPs.

*. . . The advantage for the nurses is that they have, simply because of the fact that it is written down, that it is recorded correctly, more security. . . if you make a phone call, you have to hope that it has actually arrived correctly. If I write down, for them, that I would like to have this dosage with that dosage modality, then it’s just safer and just a better support. (ID11)*

As with data from the expert interviews, data from the group interviews was additionally analyzed with regards to the specific efforts and benefits of implementing SiMbA that were mentioned. The benefits of interprofessional cooperation and communication (e.g., increased ease of work, saved time) and additional efforts (e.g., increased difficulty of work, greater time consumption) balanced each other out. Half of the interviewees perceived closer interprofessional cooperation as a positive outcome, whereas the other half perceived it as a burden that placed demands on their time.

### Conditions for the successful implementation of the three-part intervention

The following factors motivated the participants to join the project and implement the intervention in their professional routines: interest in medication safety, the opportunity to foster close interprofessional cooperation, and the emerging advantageous outcomes for their own daily work processes.

*What motivated me? Well, the interest [in the topic] and also the possibility to improve something. Now, of course, the cooperation with the pharmacy and also with the nursing home, and also to improve the medication. (ID18)*

Yes, certainly, because it started well, and because I also got a lot of information out of it and benefited from it on the road. (ID11)

The motivation to participate in SiMbA and participating professionals’ openness to technological innovation was influenced by a sense of professional ethical obligations.

*I believe this is an attitude that is simply part of the profession. That is acceptance; that is renewal and also a good development. And I believe that they are already using these resources because it’s actually a relief for everyone involved. (ID17)*

The committed participation of the various professional groups in the SiMbA-study enhanced their individual commitment and motivation to continue their involvement in the intervention.

*. . . how important it is that the three fields work together and that an exchange takes place and that everyone takes each other seriously . . . So, I think that this level has certainly changed! (ID7)*

Continuous support and extensive information played a decisive role in the successful implementation of the interventions.

*. . . the commitment and that everything was always very, well, just very well cared for. If there were problems somewhere, then it was worked on superfast. Yes, as I said, I think the direction is right . . .. So, the study certainly worked well. (ID3)*

## Discussion

The present study highlights the perceived benefits and challenges of an interprofessional HIT-intervention designed to improve medication safety in NHs. By conducting a multi-part evaluation, we were able to explore the characteristics of the impact as well as the implementation of interventional measures as perceived by those responsible in routine care.

Regarding our first research question (appropriateness of the intervention), increased vigilance about medication safety, ease of work, safety of information flow and improved interprofessional cooperation resulting in a perceived benefit for participants as well as residents point towards the usefulness of the intervention and its positive impact on work processes. However, these benefits have certain demanding prerequisites and the means for achieving them may come with unintended consequences. For example, previous research has demonstrated that HIT is able to disrupt working communication patterns between healthcare professionals [44, 45], causes issues in workflow and fosters additional work, new types of errors, overdependence on technology, paper persistence, adverse emotions [46] like stress associated with HIT usage [47] as well as an interference with work-life balance [48]. Since integrating HIT was associated with concerns about the expectation to be constantly available and affected by dislike regarding informational technology use, our results resemble some of these unintended consequences of HIT. However, the HIT-tool SiM-Pl was rather associated with time saving, whilst additional time burden was ascribed to interprofessional cooperation itself as previously reported in literature [49]. Concerning prerequisites, research has shown that successful implementation of HIT depends on its sufficient integration into existing working environments and relationships [50]. In practice, implementation of HIT is often done in an unsystematic approach [51] with insufficient consideration of technological infrastructure, support, and staff training [51, 52]. Tailoring measures to existing care practice was a principle of our intervention and its success is reflected in the respondents' positive judgement regarding the practicability of the interventional measures. Nevertheless, a problem of integration into existing working environments – the synchronization with the GPs’ and pharmacists’ documentation systems – turned out to be the most critical issue in our study. Against this background, consequently applying an implementation science approach, which suggest throughout engagement of all relevant stakeholders at the planning stage of an intervention [53], may foster (and may have fostered) the fulfilment of all prerequisites for an appropriate intervention.

Though well-known and quite prevalent, unfilled prerequisites and unintended consequences of interventions do not inherently reduce the many opportunities offered by interprofessional cooperation and HIT use. The importance of interprofessional cooperation for projects that aim to improve medication safety has been emphasized by various studies; thus, interprofessional cooperation has been promoted by implementing various measures (e.g., conducting interprofessional case discussions) [10–12, 14]. According to Pirnejad et al. (2008) and Salahuddin & Ismail (2015) [13, 14] modern communication and documentation technology (e.g., SiM-Pl) facilitate cooperation and communication. Our results support these findings in that interprofessional cooperation was associated with reduced interprofessional prejudice and improved medication safety (i.e., as perceived by those involved). Nevertheless, the present findings do not permit us to ascertain whether the positive effect on cooperation can be attributed specifically to the use of the HIT tool (i.e., SiM-Pl).

Regarding our second research question (facilitators of implementation), participants emphasized several essential facilitators for the successful implementation and prolongation of the HIT-driven intervention. Among the myriad of such conditions established in literature [52, 54], especially socio-normative factors like committed participation and mutual appreciation of the various professionals, ethical obligation, and the opportunity to foster (interprofessional) cooperation were deemed noteworthy by participants in our study. Furthermore, immediate and continuous support of the implementation process was mentioned, whilst features of the HIT-tool itself, e.g., perceived usefulness and ease of use as essential aspects according to technology acceptance models [55], were not considered. Thus, the functionality of the HIT-tool and its new features seem to have served as necessary, but not sufficient motivational ‘vehicle’ for successful integration into routine care.

This study has several limitations. First, results may be influenced by the selection bias that especially professionals interested in interprofessional cooperation or HIT participated in our study. Second, because of the project orientation taking into account the specific circumstances in the NHs (i.e. HIT, GPs, interprofessional cooperation) and the small study sample, the results of the present study should be cautiously interpreted and transferred to other NHs. Third, due to the small sample size, the systematic juxtaposition of profession-specific perspectives of GPs, pharmacists and nurses had to be omitted. The anonymity of the study participants would otherwise not have been guaranteed.

Nevertheless, the present findings can serve to guide the successful development and implementation of other interventions that aim to promote medication therapy safety through interprofessional cooperation and implementation of HIT-tools.

## Conclusion

This study shows that drug safety trainings, interprofessional drug therapy checks and a HIT-tool to enable and support interactive communication and documentation between professions involved in medication therapy are useful and feasible elements of an intervention intended to increase medication therapy safety via interprofessional cooperation. The implementation of such HIT-based measures can be effective but is prone to various pitfalls that are highlighted in the study. A critical challenge for successful implementation is the combination of both, ensuring prerequisites like training, alignment to existing care processes and usability of HIT, while anticipating new problems that arise from HIT-integration on the one hand and changes in interprofessional cooperation on the other. It is therefore essential to thoroughly evaluate interventions that are grounded in HIT-supported interprofessional cooperation.

## Supplementary Information

**Additional file 1.**

**Additional file 2.**

## Data Availability

The dataset of the System Usability Scale is available in the figshare repository, 10.6084/m9.figshare.20228163.v1, https://figshare.com/articles/dataset/System_Usability_Scale_Data/20228163. Since study participants only consented to the publication of anonymized anchor examples, full transcripts of the interviews and group discussions are not available for confidentiality reasons.

## Abbreviations

GP(s): General practitioner(s)
HIT: Health information technology
ID: Anonymization of the participants
iOS: Mobile operating system by Apple
iPad: Tablet personal computer made by Apple
MAXQDA: Software for qualitative and mixed methods data analysis
MAI: Medication appropriateness index
n: Sample size
NH(s): Nursing home(s)
NHR(s): Nursing home resident(s)
SiMbA: “Sicherheit der Medikamentherapie bei AltenheimbewohnerInnen”, Safety of medication therapy in NHRs
SiM-Pl: SiMbA-Platform
SUS: System Usability Scale
WLAN: Wireless Local Area Network
