# Supplementary material for: Evaluation of an intervention to improve the safety of medication therapy via HIT-supported interprofessional cooperation in long-term care – a mixed method study
Source: BMC Health Serv Res. 2022 Oct 3;22:1227. doi: 10.1186/s12913-022-08562-6 (PMC9531388; doi:10.1186/s12913-022-08562-6)
Supplement: Supplementary file 1 — Additional file 1. [file 12913_2022_8562_MOESM1_ESM.docx]

# Interview Schedules

## Expert interviews

Interview guide: general practitioners

| Brief introduction (ca. 2 minutes)  Welcoming   - Name, profession, role as interviewer for the evaluation of the SiMbA project.   Duration and procedure of interview   - Interview takes ca. 25–50 minutes. - Tape recording, which is transcribed anonymously; audio recording is subsequently deleted. - The interview is voluntary and can be stopped at any time without justification, the recording is then immediately deleted. - If the interviewee agrees on these terms, the interview and recording can start. |
| --- |
| Interview about SiMbA (ca. 25 – 50 minutes)   1. General impressions and experiences with the SiMbA project    1. Motivation to participate in SiMbA   You participated in the SiMbA project as a physician. We invited you by phone and letter in late summer 2016 to participate in the SiMbA project.   - What was it like when you were asked to participate in SiMbA? - What ultimately persuaded you to participate? (Probes: What convinced you to participate?) - Where did you still miss information about SiMbA, what could have been explained to you more precisely or in more detail about the SiMbA project? - What convinced you to stick with it until the end?   1. SiMbA training (face-to-face event and online training)   SiMbA provided training in which you participated when the project started. There was a face-to-face event, which was also recorded as video documentation in case you could not attend, and there was also an online training with a case study.   - How much new did you learn from the SiMbA training as a general physician? - How did you perceive the face-to-face event regarding the content and the meeting/exchange with the nurses and pharmacists? - How do you evaluate the online training (also) compared to face-to-face events? - Which mode of training on drug therapy safety would you recommend for SiMbA: face-to-face event, online event, or a mixture of both?   1. Cooperation with the other professional groups involved   As you know, drug therapy is carried out in close cooperation between the attending physicians, pharmacists and nurses.   - From your point of view, what influence has the SiMbA project had on the cooperation between the professional groups involved, i.e. you, the nurses and the pharmacists? - When you think of your responsibilities regarding ensuring drug therapy; has SiMbA had any effect on cooperation with nurses and pharmacists? If so, can you say which effect exactly? - In your opinion, what opportunities does a project like SiMbA offer to further deepen the cooperation between the professional groups involved, i.e. you, the nurses, and the pharmacists? - Independently of SiMbA and the messaging tool in SiM-PL; what do you think could be done to further promote and deepen the professional exchange between you, the pharmacists, and the nurses?   1. Communication with the other professional groups involved   As you know, exchange and communication between physicians, nurses and pharmacists is an essential part of the close cooperation in drug therapy.   - In your view, what has changed in the nature of communication, of professional exchange, as a result of the SiMbA project? - Has SiMbA had an impact on the frequency of contact between you, the nurses, and the pharmacists? - What has changed about your position as a primary care physician/family practitioner given the role of advice seeker, counselor, initiator, or facilitator in medication management since SiMbA?   (Probes: Have you been asked more often about medication by nurses or pharmacists? Have you inquired about medication with nurses or pharmacists more often? Have you been offered medication analyses (MA) by pharmacists more often? Was the result of the therapy monitoring (TBB) the reason for the MA request to the pharmacists?)   1. General experiences/appraisements with/about SiM-Pl   I would now like to ask you in general about your experiences in using SiM-Pl – since a detailed exchange about SiM-Pl will take place at the final meeting.  In the SiMbA project, you were provided with an iPad for using the SiM-Pl tool, as well as a token for secure  dial-in to the data portal. This enables you to access the electronic health record of the residents/your patients via SiM-Pl from outside the nursing care center at any time. In this way, you can view or, depending on your access rights, edit the patients'/residents' diagnoses and medications. There is also a messaging system in SiM-Pl that allows exchanges between you, the nurses, and the pharmacists.   - How useful is SiM-Pl for your everyday work? - What benefits do you see in the SiM-Pl messaging system? - With the introduction and use of SiM-PL, what changes have occurred in the frequency of contact between you, nurses, and pharmacists? - Is contact more frequent - less frequent - equally frequent as a result of the usage of SiM-Pl? - In what way could the SiM-Pl tool with the messaging system be useful/used for the cooperation between you, pharmacists and nurses? - What was your experience of using SiM-Pl with the iPad? - What would you improve about SiM-Pl, what could be optimized? - Can you say something about the use of the token for secure dial-in to SiM-Pl?  1. Appraisement of the long-term continuation and expansion of SiMbA    1. Motivation for the continuation of SiMbA  - In your opinion, what are the reasons for continuing and expanding SiMbA and SiM-Pl? - Would it be worthwhile for you personally to continue with SiMbA and SiM-Pl? - What do you think might motivate nurses and pharmacists to participate in SiMbA?   1. Recommendations for the expansion of SiMbA - In your opinion, how could your own professional group, i.e. family physicians, be motivated to participate actively and convincedly in SiMbA; what incentives should be created?   (Probes: Could the continuing education points of the certified training motivate? What impact on motivation could a provided tablet/iPad have? What other incentives should possibly be considered?   - How do you view SiMbA in the context of eMedication and ELGA?  1. Closing statement (in the sense of a short summary)  - What did you personally find good about SiMbA? - What could have been done better in SiMbA? - Is there anything else you would like to say to the SiMbA team or give them to take along? |
| Brief interview (see profile of individual interviews with general practitioners)  Finally, I would like to ask you for information about your profile:  - In which age group may I classify you:  20-29 years  30-39 years  40-49 years  50-59 years  60-69 years   - How many years have you been practicing medicine? - How many years have you been in charge of nursing homes for the elderly or geriatric care facilities? - Do you have any additional professional qualifications or further training relevant to the elderly sick and in need of care? (Which?) |
| End of interview (ca. 2 minutes)   - Then, on behalf of the SiMbA team, I would like to thank you once again for participating in SiMbA and the interview!   We will of course inform you about the results of the SiMbA study. (Farewell) |

Interview guide: nurses

| Brief introduction (ca. 2 minutes)  Welcoming   - Name, profession, role as interviewer for the evaluation of the SiMbA project.   Duration and procedure of interview   - Interview takes ca. 25–50 minutes. - Tape recording, which is transcribed anonymously; audio recording is subsequently deleted. - The interview is voluntary and can be stopped at any time without justification, the recording is then immediately deleted. - If the interviewee agrees on these terms, the interview and recording can start. |
| --- |
| Interview about SiMbA (ca. 25 – 50 minutes)   1. General impressions and experiences with the SiMbA project    1. Motivation to participate in SiMbA   You participated in the SiMbA project as a nurse. We invited you by phone and letter in late summer 2016 to participate in the SiMbA project.   - What was it like when you were asked to participate in SiMbA? - What ultimately persuaded you to participate? (Probes: What convinced you to participate?) - Where did you still miss information about SiMbA, what could have been explained to you more precisely or in more detail about the SiMbA project? - What convinced you to stick with it until the end?   1. SiMbA training (face-to-face event and online training)   SiMbA provided training in which you participated when the project started. There was a face-to-face event, which was also recorded as video documentation in case you could not attend, and there was also an online training with a case study.   - How much new did you learn from the SiMbA training as a nurse? - How did you perceive the face-to-face event regarding the content and the meeting/exchange with the general practitioners and pharmacists? - How do you evaluate the online training (also) compared to face-to-face events? - Which mode of training on drug therapy safety would you recommend for SiMbA: face-to-face event, online event, or a mixture of both?      - 1. Cooperation with the other professional groups involved   As you know, drug therapy is carried out in close cooperation between the attending physicians, pharmacists and nurses.   - From your point of view, what influence has the SiMbA project had on the cooperation between the professional groups involved, i.e. you, the general practitioners and the pharmacists? - When you think of your responsibilities regarding ensuring drug therapy; has SiMbA had any effect on cooperation with general practitioners and pharmacists? If so, can you say which effect exactly? - In your opinion, what opportunities does a project like SiMbA offer to further deepen the cooperation between the professional groups involved, i.e. you, the general practitioners, and the pharmacists? - Independently of SiMbA and the messaging tool in SiM-PL; what do you think could be done to further promote and deepen the professional exchange between you, the pharmacists, and the general practitioners?   1. Communication with the other professional groups involved   As you know, exchange and communication between physicians, nurses and pharmacists is an essential part of the close cooperation in drug therapy.   - In your view, what has changed in the nature of communication, of professional exchange, as a result of the SiMbA project? - Has SiMbA had an impact on the frequency of contact between you, the general practitioners, and the pharmacists? - What has changed about your position as a nurse given the role of advice seeker, counselor, initiator, or facilitator in medication management since SiMbA?   (Probes: Have you been asked more often about medication by general practitioners or pharmacists? Have you inquired about medication with general practitioners or pharmacists more often? Have you been offered medication analyses (MA) by pharmacists more often? Was the result of the therapy monitoring (TBB) the reason for inquiries by the general practitioner or MA requests to the pharmacists?)   1. General experiences/appraisements with/about SiM-Pl   I would now like to ask you in general about your experiences in using SiM-Pl – since a detailed exchange about SiM-Pl will take place at the final meeting.  In the SiMbA project, you were provided with an iPad for using the SiM-Pl tool, as well as a token for secure  dial-in to the data portal. This enables you to access the electronic health record of the residents/your patients via SiM-Pl from outside the nursing care center at any time. In this way, you can view or, depending on your access rights, edit the patients'/residents' diagnoses and medications. There is also a messaging system in SiM-Pl that allows exchanges between you, the general practitioners, and the pharmacists.   - How useful is SiM-Pl for your everyday work? - What benefits do you see in the SiM-Pl messaging system? - With the introduction and use of SiM-PL, what changes have occurred in the frequency of contact between you, nurses, and pharmacists? - Is contact more frequent - less frequent - equally frequent as a result of the usage of SiM-Pl? - In what way could the SiM-Pl tool with the messaging system be useful/used for the cooperation between you, pharmacists and general practitioners? - What was your experience of using SiM-Pl with the iPad? - What would you improve about SiM-Pl, what could be optimized? - Can you say something about the use of the token for secure dial-in to SiM-Pl?  1. Appraisement of the long-term continuation and expansion of SiMbA    1. Motivation for the continuation of SiMbA  - In your opinion, what are the reasons for continuing and expanding SiMbA and SiM-Pl? - Would it be worthwhile for you personally to continue with SiMbA and SiM-Pl? - What do you think might motivate general practitioners and pharmacists to participate in SiMbA?   1. Recommendations for the expansion of SiMbA - In your opinion, how could your own professional group, i.e. nurses, be motivated to participate actively and convincedly in SiMbA; what incentives should be created?   (Probes: Could the approval of the certified training as continued education motivate? What other incentives should possibly be considered?)   - How do you view SiMbA in the context of eMedication and ELGA?  1. Closing statement (in the sense of a short summary)  - What did you personally find good about SiMbA? - What could have been done better in SiMbA? - Is there anything else you would like to say to the SiMbA team or give them to take along? |
| Brief interview (see profile of individual interviews with general practitioners)  Finally, I would like to ask you for information about your profile:  - In which age group may I classify you:  20-29 years  30-39 years  40-49 years  50-59 years  60-69 years   - How many years have you been a nurse? - How many years have you been working in nursing homes for the elderly or geriatric care facilities? - Do you have any additional professional qualifications or further training relevant to the elderly sick and in need of care? (Which?) |
| End of interview (ca. 2 minutes)   - Then, on behalf of the SiMbA team, I would like to thank you once again for participating in SiMbA and the interview!   We will of course inform you about the results of the SiMbA study. (Farewell) |

Interview guide: pharmacists

| Brief introduction (ca. 2 minutes)  Welcoming   - Name, profession, role as interviewer for the evaluation of the SiMbA project.   Duration and procedure of interview   - Interview takes ca. 25–50 minutes. - Tape recording, which is transcribed anonymously; audio recording is subsequently deleted. - The interview is voluntary and can be stopped at any time without justification, the recording is then immediately deleted. - If the interviewee agrees on these terms, the interview and recording can start. |
| --- |
| Interview about SiMbA (ca. 25 – 50 minutes)   1. General impressions and experiences with the SiMbA project    1. Motivation to participate in SiMbA   You participated in the SiMbA project as a pharmacist. We invited you by phone and letter in late summer 2016 to participate in the SiMbA project.   - What was it like when you were asked to participate in SiMbA? - What ultimately persuaded you to participate? (Probes: What convinced you to participate?) - Where did you still miss information about SiMbA, what could have been explained to you more precisely or in more detail about the SiMbA project? - What convinced you to stick with it until the end?   1. SiMbA training (face-to-face event and online training)   SiMbA provided training in which you participated when the project started. There was a face-to-face event, which was also recorded as video documentation in case you could not attend, and there was also an online training with a case study.   - How much new did you learn from the SiMbA training as a pharmacist? - How did you perceive the face-to-face event regarding the content and the meeting/exchange with the general practitioners and nurses? - How do you evaluate the online training (also) compared to face-to-face events? - Which mode of training on drug therapy safety would you recommend for SiMbA: face-to-face event, online event, or a mixture of both?   1. Cooperation with the other professional groups involved   As you know, drug therapy is carried out in close cooperation between the attending physicians, pharmacists and nurses.   - From your point of view, what influence has the SiMbA project had on the cooperation between the professional groups involved, i.e. you, the general practitioners and the nurses? - When you think of your responsibilities regarding ensuring drug therapy; has SiMbA had any effect on cooperation with general practitioners and nurses? If so, can you say which effect exactly? - In your opinion, what opportunities does a project like SiMbA offer to further deepen the cooperation between the professional groups involved, i.e. you, the general practitioners, and the nurses? - Independently of SiMbA and the messaging tool in SiM-PL; what do you think could be done to further promote and deepen the professional exchange between you, the nurses, and the general practitioners?   1. Communication with the other professional groups involved   As you know, exchange and communication between physicians, nurses and pharmacists is an essential part of the close cooperation in drug therapy.   - In your view, what has changed in the nature of communication, of professional exchange, as a result of the SiMbA project? - Has SiMbA had an impact on the frequency of contact between you, the general practitioners, and the nurses? - What has changed about your position as a pharmacist given the role of advice seeker, counselor, initiator, or facilitator in medication management since SiMbA?   (Probes: Have you been asked more often about medication by general practitioners or nurses? Have you inquired about medication with general practitioners or nurses more often? Have you been asked for medication analyses (MA) by nurses or general practitioners more often?)   1. General experiences/appraisements with/about SiM-Pl   I would now like to ask you in general about your experiences in using SiM-Pl – since a detailed exchange about SiM-Pl will take place at the final meeting.  In the SiMbA project, you were provided with an iPad for using the SiM-Pl tool, as well as a token for secure  dial-in to the data portal. This enables you to access the electronic health record of the residents/your patients via SiM-Pl from outside the nursing care center at any time. In this way, you can view or, depending on your access rights, edit the patients'/residents' diagnoses and medications. There is also a messaging system in SiM-Pl that allows exchanges between you, the general practitioners, and the nurses.   - How useful is SiM-Pl for your everyday work? - What benefits do you see in the SiM-Pl messaging system? - With the introduction and use of SiM-PL, what changes have occurred in the frequency of contact between you, nurses, and pharmacists? - Is contact more frequent - less frequent - equally frequent as a result of the usage of SiM-Pl? - In what way could the SiM-Pl tool with the messaging system be useful/used for the cooperation between you, nurses, and general practitioners? - What was your experience of using SiM-Pl with the iPad? - What would you improve about SiM-Pl, what could be optimized? - Can you say something about the use of the token for secure dial-in to SiM-Pl?  1. Appraisement of the long-term continuation and expansion of SiMbA    1. Motivation for the continuation of SiMbA  - In your opinion, what are the reasons for continuing and expanding SiMbA and SiM-Pl? - Would it be worthwhile for you personally to continue with SiMbA and SiM-Pl? - What do you think might motivate general practitioners and nurses to participate in SiMbA?   1. Recommendations for the expansion of SiMbA - In your opinion, how could your own professional group, i.e. pharmacists, be motivated to participate actively and convincedly in SiMbA; what incentives should be created?   (Probes: Could the continuing education points of the certified training motivate? What impact on motivation could a provided tablet/iPad have? What other incentives should possibly be considered?)   - How do you view SiMbA in the context of eMedication and ELGA?  1. Closing statement (in the sense of a short summary)  - What did you personally find good about SiMbA? - What could have been done better in SiMbA?   Is there anything else you would like to say to the SiMbA team or give them to take along? |
| Brief interview (see profile of individual interviews with general practitioners)  Finally, I would like to ask you for information about your profile:  - In which age group may I classify you:  20-29 years  30-39 years  40-49 years  50-59 years  60-69 years   - How many years have you been a pharmacist? - How many years have you been in charge of nursing homes for the elderly or geriatric care facilities? - Do you have any additional professional qualifications or further training relevant to the elderly sick and in need of care? (Which?) |
| End of interview (ca. 2 minutes)   - Then, on behalf of the SiMbA team, I would like to thank you once again for participating in SiMbA and the interview!   We will of course inform you about the results of the SiMbA study. (Farewell) |

## Focus group discussions

Interview guide: group discussion

| Hardware rating: iPad |
| --- |
| Cognitive perspective (Slide)  This is about: Applicability, technical usability of the iPad. |
| You had been provided with an iPad for accessing SiM-PL or PflegeDoku-Mobil. Please take another look at the iPad as a technical commodity ... also in view of the technical usability and functionality!   - What is your impression of the usability and functions of the iPad?   (Probes: What did you particularly like about the iPad's usability?)   - Where did you encounter problems with the iPad device?   (Probes: What could be better about the iPad device?)   - What features were missing or should be different?   (Probes: e.g. jacks, charger, USB adapters for other technical/electronic devices, external keyboard, integrated USB adapters?)  You were able to access SiM-Pl or PflegeDoku-Mobil via iPad or via your PC.   - How did you find working in SiM-PL or PflegeDoku-Mobil with the iPad compared to your PC?   (Probes: Where did you find working with the iPad better? Where did you find the PC better?) |
| Motivational perspective (Slide)  This is about: Influence of the iPad on behavior in everyday work, attitude toward work design. |
| - What influence does the iPad have …   … on your everyday work  … on your work processes?  (Probes: Can you think of anything else when you think of the fact that with the iPad you can…  … as a doctor or pharmacist; access the documentation of your patients/residents at any time of the day from any location?  … as nurse; access the EHR not only in the office but directly in the immediate vicinity of the residents?   - What influence does the iPad have on your attitude …   … regarding the direct and prompt documentation of orders, changes in therapy, progress, etc.?  ... regarding retrieval of information about your residents? |
| Emotional perspective (Slide)  This is about: Aesthetics, aesthetic sensation when using the iPad). |
| It is now just a matter of judging the iPad purely visually as an object of everyday (work) life.   - What do you like about the iPad, what is really beautiful?   (Probes: What should a portable computing device/tablet look like so that you find it beautiful?  Also think about color, size, grip, protective case, etc.)   - What do you not like at all about the iPad, what do you find ugly? |
| Experiential, hedonic perspective (Slide)  This is about: Enhancing personal well-being by working with the iPad |
| - What do you see as the biggest benefit of the iPad …   … for your professional everyday life?  … for your private everyday life?  (Probes: Can you think of any other advantages or disadvantages when you think about the fact that you can use the iPad to access the documentation of your patients/residents at any time of day, at any location or in the immediate vicinity of the residents, even away from the office?)  You can also use other technical functions of the iPad (Internet, camera, etc.).   - Given its many technical possibilities:   … what pleases you, makes you really enjoy the iPad?  … what bothers you, what annoys you, is useless about the iPad? |

| Hardware rating: Token |
| --- |
| Cognitive perspective (Slide)  This is about: Applicability, technical usability of the Token. |
| You had been provided with an iPad for accessing SiM-PL or PflegeDoku-Mobil. Please take another look at the iPad as a technical commodity ... also in view of the technical usability and functionality!   - What is your impression of the usability of the synchronized (functional) token?   (Probes: What did you particularly like about the token?)   - What could be better about the token?   (Probes: Where have there been problems with the token?) |
| Motivational perspective (Slide)  This is about: Influence of the token on behavior in everyday work, attitude toward work design. |
| - What influence does the token have on your motivation to dial into SiM-Pl?   (Probes: What motivates you about the token to dial into SiM-PL ... what demotivates you)? |
| Emotional perspective (Slide)  This is about: Aesthetics, aesthetic sensation when using the iPad). |
| It is now just a matter of judging the iPad purely visually as an object of everyday (work) life.   - What do you really like about the token?   (Probes: What should the token look like so that you find it beautiful?  Also think about color, size, grip, protective case, etc.)   - What do you not like at all about the token, what do you find ugly? |
| Experiential, hedonic perspective (Slide)  This is about: Enhancing personal well-being by working with the iPad |
| - What do you see as the biggest benefit of the token …   … for your professional everyday life?  … for your private everyday life? |

| Evaluation of the program: SiM-Pl/PflegeDoku-Mobil |
| --- |
| Cognitive perspective (Slide)  This is about: Applicability, technical usability of the documentation program. |
| You access the SeneCura nursing documentation program via SiM-PL/PflegeDoku-Mobil; it has the same structure as the documentation program with which you work via the nursing care center at the facility.   - What is your impression of the usability of the documentation program SiM-PL/PflegeDoku-Mobil?   (Probes: What did you particularly like about the program functions?)   - What could be better about the documentation program?   (Probes: Where did you have difficulties, for example, when retrieving or entering resident data?)   - Which features and additional functions were missing in the documentation program or should be different?   (Probes: e.g. popups/info on individual input fields, filter functions, free text fields, links to other fields, copy-paste functions)  SiM-PL and PflegeDoku-Mobil also provide a message system for online communication.   - What is your impression of this messaging system?   (Probes: What did you like most about the messaging system?   - What could be better about the messaging system?   (Probes: Where did you encounter problems when using the messaging system?)   - What additional functions should the messaging system have?   (Probes: e.g. links, possibility for file attachments)? |
| Motivational perspective (Slide)  This is about: Influence of the iPad on behavior in everyday work, attitude toward work design. |
| You can use SiM-PL/PflegeDoku-Mobil to directly access areas of the nursing documentation that are important to you and you can make all entries directly yourself (i.e. from outside or outside the nursing office).   - What influence does this direct access/documentation possibility have?   ... on your everyday work and on work processes?  ... on documentation (e.g. orders, changes in therapy, progress)  ... on the retrieval of resident data and information? (Probe: To get an overview)  SiM-PL/PflegeDoku-Mobil also offers you a messaging portal for the exchange of messages; for online exchange among each other.   - How much do you like exchanging messages with colleagues from other professional groups via the portal? - What influence does the messaging portal of SiM-PL/PflegeDoku-Mobil have on your desire to get into contact and exchange with colleagues of other professional groups?   (Probes: What motivates you to use the messaging system ... what demotivates you?) |
| Emotional perspective (Slide)  This is about: Aesthetics, aesthetic sensation when using the iPad). |
| It is now just a matter of judging the documentation program visually as an object of everyday (work) life.   - What do you like about the documentation program, what is really beautiful?   (Probes: What should a documentation system look like so that you find it beautiful?  Also think about color, size, grip, protective case, etc.)   - What do you not like at all about the documentation system, what do you find ugly?   Now it is about aesthetics and sensations when using the messaging system:   - What do you really like about the messaging system?   (Probes: What should a messaging system look like so that you find it beautiful?  Also think about color, size, grip, protective case, etc.)  What do you not like at all about the messaging system, what do you find ugly? |
| Experiential, hedonic perspective (Slide)  This is about: Enhancing personal well-being by working with the iPad |
| You can use the iPad to access your patients'/residents' data at any time of day, at any location or in the immediate vicinity of the residents, even away from the care center via using SiM-PL/PflegeDoku-Mobil.  In addition, other functions linked to the documentation program can be used (e.g. copy function, camera, etc.).   - Given its many technical possibilities:   … what pleases you, makes you really enjoy the program?  … what bothers you, what annoys you, is useless about the program?   - What do you see as the biggest benefit of the documentation program:   … for your professional everyday life?  … for your private everyday life? |
