# Supplementary material for: Evaluation of an intervention to improve the safety of medication therapy via HIT-supported interprofessional cooperation in long-term care – a mixed method study
Source: BMC Health Serv Res. 2022 Oct 3;22:1227. doi: 10.1186/s12913-022-08562-6 (PMC9531388; doi:10.1186/s12913-022-08562-6)
Supplement: Supplementary file 2 — Additional file 2. [file 12913_2022_8562_MOESM2_ESM.docx]

# Risks and counterstrategies regarding telephone interviews

When compared to face-to-face interviews, telephone interviews are more advantageous because they are more time and cost effective and offer better access to potential participants [1, 2, 3]. Telephone surveys are also associated with lower interview effects/influences and better control, which in turn result in lower response homogeneity (i.e., when compared to face-to-face interviews). However, a prerequisite for this is a limited number of interviews per interviewer (i.e., a maximum of 15 interviews) [2].

|  | Risks associated with telephone surveys | Counterstrategies |
| --- | --- | --- |
| Data quality | - Risk of social desirability and “satisficing” of the interviewer [3]  - Everyday telephone conversations differ from face-to-face conversations in the length of the dialogue; this influences the data quality of telephone interviews in comparison to face-to-face interviews (e.g., participants answer too quickly or provide brief responses; lower levels of patience on the part of the interviewers; they might not wait long enough for possible clarifications, move on to the next question very quickly; rarely use probes to further understand the disclosed material]) [1] | - Employ experienced interviewers who are not involved in the project and are unknown to the participants  - Train interviewers  - Emphasize the importance of probing/probes  - Multiple pretests, joint evaluation of these test interviews |
| Homogenization | Too many (>15 interviews) lead to interviewer fatigue, resulting in interviews that are too similar [3] | - Assign a maximum of 14 interviews to an interviewer |
| Nonverbal communication | No control over the interaction or the course of the conversation (e.g. it is not clear whether or how an interviewee becomes involved in the interview and withdraws from the conversation or whether the situation or questions are unpleasant [1, 4]. Moreover, nonverbal information cannot be recorded [4]. | - Employ experienced interviewers  - Train interviewers  - Emphasize the importance of probing/probes  - Multiple pretests, joint evaluation of these test interviews |

Supplementary table S1: The risk of telephone surveys and counterstrategies

Hedonic-pragmatic model

| The principles of the usability of the SiM-Pl  (Cf. the hedonic-pragmatic model of Diefenbach & Hassenzahl (2017) [5] |
| --- |
| Cognitive (pragmatic) perspective  - Practicability of the hardware and software of the SiM-Pl tool  Motivational perspective  - Influence of the use of the SiM-Pl on user behaviors and attitudes  Emotional perspective  - Users’ perceptions of the aesthetic appeal of the SiM-Pl.  Experience-oriented, hedonic perspective  - An improvement in the well-being of users because of the use of the SiM-Pl |

Supplementary table S2: The principles of the usability of the SiM-Pl in accordance with Diefenbach & Hassenzahl’s (2017) hedonic-pragmatic model [5].

# Reference list

1. Rubin HJ, Rubin IS. Qualitative Interviewing: The Art of Hearing Data. 2 ed. Tausend Oaks, London, Dehli: Sage Publications, Inc.; 2005.

2. Diefenbach S, Hassenzahl M. Psychologie in der nutzerzentrierten Produktgestaltung. Mensch-Technik-Interaktion-Erlebnis. Brodbeck FC, Kichler E, Woschée R, editors. Berlin: Springer-Verlag GmbH; 2017.

3. Glantz A, Michael T. Intervieweffekte. In: Bauer N, Blasius J, editors. Handbuch Methoden der empirischen Sozialforschung. Wiesbaden: Springer VS Fachmedien; 2014. p. 313-322.

4. Huefken V. Telefonische Befragung. In: Bauer N, Blasius J, editors. Handbuch Methoden der empirischen Sozialforschung. Wiesbaden: Springer VS Fachmedien; 2014. p. 631-641.

5. Novick G. Is there a bias against telephone interviews in qualitative research? Research in nursing & health. 2008;31(4):391-398.
